# “MacCallum Plaque of the Heart”: A Medicolegal Case

**DOI:** 10.4103/1995-705X.73220

**Published:** 2010

**Authors:** Udasimath Shivakumarswamy, Sankappa P. Sinhasan, R. Purushotham, K. R. Nagesha

**Affiliations:** Department of Pathology, Hassan Institute of Medical Sciences, Karnataka, India

**Keywords:** MacCallum plaques, mitral valve vegetations, rheumatic heart disease

## Abstract

Mural endocardial lesions can be seen as MacCallum plaques in rheumatic heart disease. These plaques appear as map-like areas of thickened, roughened, and wrinkled part of the endocardium in the left atrium. Perhaps they are caused by regurgitant jets of blood flow, due to incompetence of the mitral valve. Although MacCallum plaques are one of the characteristic features in rheumatic heart disease, they are very uncommon in recent times. We hereby report a case of an adolescent female with RHD, who was working as a housemaid in a doctor’s house for a few months, and suddenly developed respiratory tract infection and cardiac failure. She died on the fourth day of admission. A medicolegal autopsy was conducted, as her relatives accused her master of sexual assault. On autopsy it was seen that the mitral valves were narrowed, showing multiple vegetations. MacCallum plaque was seen in the dilated left atrium. Hence, it is presented here for educative purposes.

## INTRODUCTION

In India and other developing countries, rheumatic fever (RF) and rheumatic heart disease (RHD) continue to be major public health problems and contribute to significant cardiac morbidity and mortality.[1] RF is an inflammatory disease seen in children, which occurs following Group A streptococcal infection.[2] RHD is the cardiac manifestation of RF. Mural endocardial lesions can be seen as MacCallum plaques in RHD.[1,2]

About 60% of acute RF patients with cardiac failure, develop valvular diseases 10 years later.[3] They have a greater chance of improvement or even regression of the cardiac lesion if they undergo adequate secondary prophylaxis.[4]

## CASE REPORT

An adolescent female, about 18 years of age, with RHD, was working as a housemaid in a doctor’s house for a few months. She or her parents did not reveal her disease status to her employer in spite of knowing it before hand. She was on irregular treatment with no improvement. One day she suddenly developed respiratory tract infection and was hospitalized. She was diagnosed as a case of RHD complicated with heart failure. She died on the fourth day of admission. A medicolegal autopsy was conducted as her relatives accused her master of sexual assault and demanded heavy compensation.

### Autopsy findings

**Figure 1:**
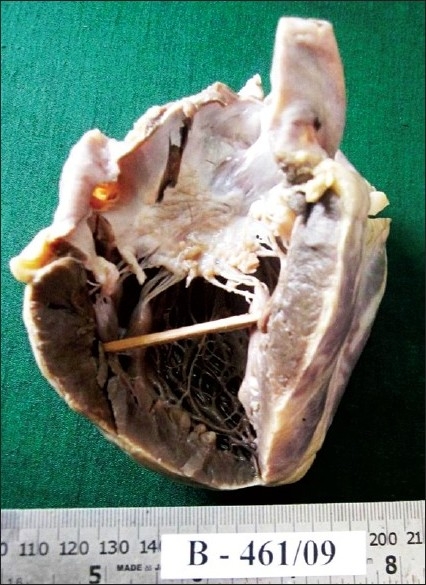
Gross appearance of heart showing dilated left atrium with MacCallum plaque and vegetations

On opening the pleural and pericardial cavities, about one liter of pus was drained. The heart weighed 282 g. The left atrium showed dilatation, with a map-like thickened, roughened, and wrinkled area in the posteromedial surface [Figure 1], which was subsequently identified as a MacCallum plaque The mitral valve showed narrowing and diffuse thickening, with a fish- mouth appearance, and multiple vegetations hanging into the left ventricle. Tendinous cords showed shortening, thickening, and fusion. The tricuspid and aortic valves were unremarkable. The left ventricle wall showed thickening.

**Figure 2:**
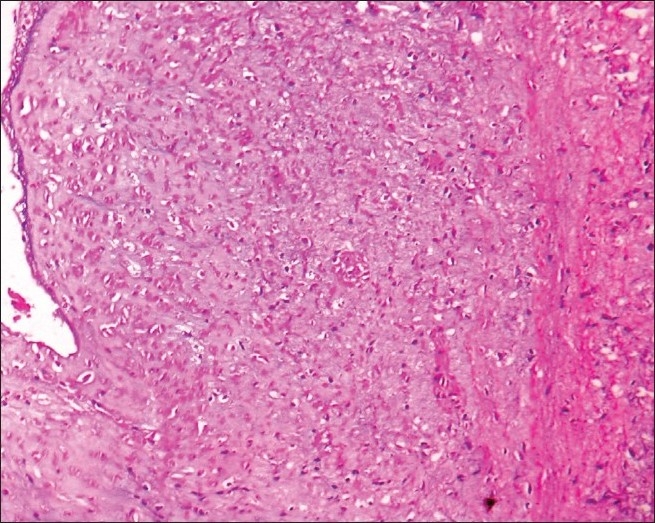
Photomicrograph of the MacCallum plaque showing diffuse subendocardial fibrosis. (H and E, 10× magnifications)

**Figure 3:**
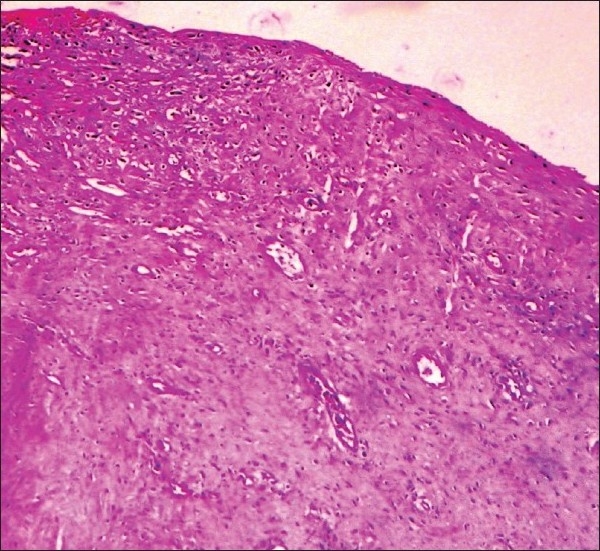
Photomicrograph of the adjacent areas of the MacCallum plaque showing mixed inflammatory cell infiltrate, with focal areas of interstitial edema and neovascularization (H and E, 10× magnifications)

Histopathological examination of multiple sections from the thickened and fibrosed area in the left atrium revealed diffuse subendocardial fibrosis [Figure 2]; the area adjacent to the fibrosis showed mixed inflammatory cell infiltrate, with focal areas of interstitial edema and neovascularization [Figure 3]. These were characteristic features of MacCallum plaques. The left ventricular wall showed diffuse hypertrophy of the cardiac muscle fibers with interstitial fibrosis.

## DISCUSSION

In India and other developing countries, RF is common and is responsible for many cases of damaged heart valves. In Western countries, it has become fairly rare since the 1960s, probably due to the widespread use of antibiotics to treat streptococcal infections.[5] Acute RF follows 3% of the cases of group A b-hemolytic streptococcal pharyngitis in children. The illness typically develops two to three weeks after a pharyngeal streptococcal infection. The disease is more severe in females than in males. Although the disease seldom occurs, it is serious and has a mortality of 2 – 5%.[2,5]

Rheumatic fever is a systemic, poststreptococcal, nonsuppurative inflammatory disease, principally affecting the heart, joints, central nervous system (CNS), skin, and subcutaneous tissues.[6] William Boyd years ago gave the dictum, “Rheumatism licks the joint, but bites the whole heart”. RF affects the periarterial connective tissue. It is believed to be caused by antibody cross-reactivity, which is a Type II hypersensitivity reaction and is termed as, ‘molecular mimicry’.[1,2,6] Cross-reactivity between cardiac myosin and group A-β hemolytic Streptococcal M protein has been adequately demonstrated.[1,2,5]

Rheumatic heart disease is the major cause of morbidity from rheumatic fever. Acute RHD often produces pancarditis, characterized by endocarditis, myocarditis, and pericarditis.[5] The inflammation may cause serofibrinous pericardial exudates described as bread-and-butter pericarditis, which usually resolves without sequelae. Endocarditis is manifested as valve insufficiency. The mitral valve is most commonly and severely affected in 65 – 70% of the patients.[2,5] Involvement of the endocardium typically results in fibrinoid necrosis and small (1 to 2 mm) vegetation-verrucae formations along the lines of closure of the mitral valves.[7] Warty projections arise from the precipitation at the sites of the erosion, related to the underlying inflammation and collagen degeneration.[2,7]

Subendocardial lesions may induce irregular thickening called MacCallum plaques, usually in the posterior wall of the left atrium just above the posterior leaflet of the mitral valve. Perhaps MacCallum plaques are caused by regurgitant jets of blood flow due to incompetence of the mitral valve.[2,5,7]

The chronic stage of RF is characterized by pancarditis, causing major cardiac sequelae, referred to as RHD, with permanent deformity of one or more valves. A chronic healed mitral valve shows ‘fish mouth’ or ‘buttonhole’ stenosis.[2,6] Chronic RHD is characterized by the organization of acute inflammation and subsequent fibrosis. The cardinal anatomic changes of the valve include leaflet thickening, commissural fusion and shortening, and thickening of the tendinous cords.[1,2,7]

A characteristic feature of the microscopic examination of acute RF is the presence of Aschoff bodies consisting of swollen eosinophilic collagen surrounded by lymphocytes, occasional plasma cells, and plump macrophages, called Anitschkow cells. These Anitschkow cells have abundant cytoplasm and central round-to-ovoid nuclei in which the chromatin is disposed in a central, slender, wavy ribbon (hence the designation ‘caterpillar cells’). Some of these become multinucleated to become Aschoff giant cells. These bodies can be found in any of the three layers of the heart. A microscopic examination of chronic RHD shows diffuse fibrosis and often neovascularization that obliterates the originally layered, avascular leaflet architecture. Aschoff bodies are replaced by a fibrous scar; therefore, diagnostic forms of these lesions are rarely seen in surgical specimens or autopsy tissues of patients with chronic RHD.[2,5]

Once the heart is involved it is often associated with reactivation and recurrence of the disease. Myocarditis in particular is the most life-threatening complication, due to involvement of the conduction system of the heart and results in serious arrhythmias.[8] Major causes of death in RHD are cardiac failure, bacterial endocarditis, and embolism.[5,8]

## CONCLUSION

Prevention of RHD is achieved by eradicating the acute infection and prophylaxis with antibiotics to decrease mortality and morbidity. Primary care physicians and nurses also play a role in the prevention, primarily in screening school-going children for sore throats that may be caused by Group A β-Hemolytic *Streptococcus pyogenes.*

One of the characteristic features of rehumatic heart disease on histology is the finding of MacCallum’s plaques. To demonstrate MacCallum’s plaques, multiple sections from thickened and fibrosed areas, especially from the left atrium should be obtained on histopathological examination.

## References

[1] Chopra P, Gulwani H (2007). Pathology and pathogenesis of rheumatic heart disease. Indian J Pathol Microbiol.

[2] Burns DK, Kumar V, Kumar (2007). The Heart. Robbins Basic Pathology.

[3] Meira ZM, Mota CD, Tortelli E, Nunan EA, Mitre AM, Moreira NS (1993). Evaluation of secondary prophylactic schemes, based on Benzathine Penicillin G, for rheumatic fever in children. J Pediat.

[4] Tompkins DG, Boxerbaum B, Liebman J (1972). Long-term prognosis of rheumatic fever patients receiving regular intramuscular Benzathine Penicillin. Circulation.

[5] Jarallah A Rheumatic heart disease is the most serious complication of rheumatic fever.

[6] Gilbert-Bareness E, Gilbert-Bareness E (2007). Cardiovascular disorders. Potter’s pathology of the fetus, infant and child.

[7] McMannus BM, Davies MJ, Damjanov I (2009). Heart disease in the adult. Anderson’s Patholgy.

[8] Akintunde AA, Opadijo OG (2009). Late presentation of rheumatic heart disease: A justification for renewal of preventive methods?. Pan Afr J.

